# Advancing healthcare worker safety in an academic hospital setting: a mixed methods quality improvement initiative protocol

**DOI:** 10.3389/frhs.2026.1676416

**Published:** 2026-05-01

**Authors:** Seema Sharma, Lee de Bie, Carrie Fletcher, Myanca Rodrigues, Brianna Depestel, Brooke Cowell, Sahar Monzavi-Bacon, Christina Bowman, Adam J. Prieur, Charlie Puma, Kwasi Adu-Basowah, Carly Weeks, Christian Rabbat, Lehana Thabane, Mike Heenan

**Affiliations:** 1 Department of Education & Learning, St Joseph's Healthcare Hamilton, Hamilton, ON, Canada; 2 Department of Health Research Methods, Evidence and Impact, McMaster University, Hamilton ON, Canada; 3 Faculty of Health Sciences, McMaster University, Hamilton ON, Canada; 4 School of Nursing, McMaster University, Hamilton, ON, Canada; 5 Department of Medicine, McMaster University, Hamilton, ON, Canada

**Keywords:** healthcare workers, incident reporting, quality improvement, safety culture, workplace violence (WPV)

## Abstract

**Background:**

There is growing evidence that workplace violence (WPV) including physical, verbal, psychological, racial and sexual violence against healthcare workers (HCW) is a globally increasing burden, with serious negative effects on the wellbeing of healthcare workers and deleterious outcomes for patients and healthcare systems. Many healthcare systems have put in place some evidence-based programs to combat WPV to provide the most safe and supportive environment while providing the highest quality, safe and compassionate care to their communities. However, the incidence of WPV continues to escalate.

**Objectives:**

The aim of this paper is to describe the methodology for quality improvement initiative to advance safety culture, by addressing WPV at St Joseph's Healthcare Hamilton (SJHH), an academic Health Sciences Centre which is part of the St Joseph's Health System in Ontario, Canada. The objectives are to: (i) assess barriers and facilitators to physician reporting of WPV incidents; (ii) evaluate gaps in existing WPV data and reporting systems; (iii) identify system-level opportunities to improve WPV prevention and response; and (iv) monitor changes in WPV related outcomes over time, including reporting rates, incident trends, and priorities identified through staff engagement.

**Methods:**

We set up the Workplace Safety Governance Committee as an advisory body to champion our strategy against WPV. Building on the work of an operational Prevention of Violence in the Workplace Committee and relying on a set of guiding principles, the Committee will use a multi component quality improvement approach informed by mixed methods that includes: (i) conducting a scoping review on physician reporting of WPV incidents; (ii) participating in an audit conducted by the Institute of Healthcare Improvement of our current practices and structures and to identify areas for improvement; (iii) engaging with provincial Workplace Safety and Insurance Board regarding mental stress injuries at SJHH, including a comparison with similar organizations; (iv) doing ‘rounds' in different hospital units to elicit concerns and advance open communication about WPV, to generate ideas for solutions and to provide regular updates and communication to share progress updates on the Committee work.

**Outcomes and analysis:**

We will use descriptive statistics and process charts to display trends over time in order to monitor changes and progress on different types of WPV related outcomes. We will also use qualitative descriptions to capture themes from the scoping review and audit.

**Discussion:**

WPV against HCW is a major barrier to achieving the goal of better health outcomes for patients and HCW. As part of the organization's 2024–25 priorities, SJHH is committed to fostering a physically and psychologically safe environment for our healthcare workers, volunteers and learners. Our quality improvement initiative consists of robust methodological approach using mixed evidence-based methods for data collection from different sources, including a survey of the literature, engagement of external stakeholder expertise on WPV and a review of our current practices and standards.

## Background

There is mounting evidence that workplace violence (WPV) against healthcare workers (HCW) is a globally increasing burden for individuals and healthcare systems alike (1–3). A recent systematic review shows the high prevalence rate of WPV of 58.7% (95% confidence interval CI: 48.51–68.92%) in hospital and prehospital settings around the world (4). WPV is generally defined as any act of physical aggression (e.g., pushing, punching, scratching, spitting, hitting, kicking, using a weapon, throwing objects, or other forms of physical harm), verbal abuse (e.g., threats, racial insults, shouting, or derogatory language), psychological abuse (e.g., intimidation or coercive behavior), or sexual harassment or assault (e.g., unwanted advances or inappropriate behavior) directed toward healthcare workers at work or while on duty (4, 5). HCW is defined as anyone who performs work in a healthcare setting who has the potential for direct or indirect exposure to patients (1, 2, 4). These include ancillary clinical staff, nurses, physicians, public health personnel, researchers, learners, non-clinical staff, aides, volunteers, laboratory technicians, or medical waste handlers. Workplace is defined as “any land, premises, location or thing at, upon, in or near which a worker works” (3) examples include a hospital, long-term care home, patient or client home while the worker is present, or worker's vehicle while used for work purposes. WPV is one of the leading forms of occupational injury among HCW with verbal violence as the leading type of WPV with a prevalence of 66.8% (95% CI: 60.96–72.56%) in hospital and pre-hospital settings globally (4, 6). Most common WPV incidents are often perpetrated by patients, families, or visitors, the very groups for whom HCW are sworn to care (7).

The negative effects of WPV on HCW are both physical and psychological (1, 8). There is robust evidence that WPV is also associated with medical errors and diminished patient safety largely due to increased staff turnover and subsequent workforce shortages (1, 8). WPV including physical, verbal, racial and sexual violence against HCW, including learners, has become an increasing burden for individuals, leading to negative patient outcomes, medical errors and harm to wellbeing, which in turn impacts healthcare systems in terms of increased costs and resource utilization (9).Furthermore, WPV may have a spillover effect beyond workplace, HCW who experience WPV are more likely to display heightened stress, which can extend to their personal lives contributing to domestic conflict or violence because stressed individuals are likely to carry feelings of anger and frustration toward people around them, which often leads to hostile or toxic environment (10). Research shows that WPV is pervasive and increasing in severity and frequency of incidents, thus raising serious concerns among healthcare system leaders and in society at large (11, 12). The underlying reasons for this surge are unclear, but may be related, in part, to the impact of COVID-19 pandemic one of the most stressful recent events that created immense psychological pressure on HCWs and the public alike and divisions in society instigated by polarized political environments around the world (13, 14). There is also growing concern that most WPV incidents go unreported for many reasons that include the feeling that WPV is “part of the job”, fear of retribution by patients or their families, guilt for reporting on patients who are already suffering with some illness and perceived lack of institutional supports etc (15–17). The greatest challenge with nonreporting or underreporting of WPV incidents is that this makes it impossible to estimate the true incidence or prevalence of the problem, creating difficulty in finding solutions.

There is considerable interventional research and resources describing various programs on how to prevent and mitigate the effects of WPV, but they have had mixed success (2, 18–22). Many healthcare systems have implemented different evidence-based educational programs such as risk recognition, communication strategies, de-escalation training/techniques, situational awareness, personal protective measures, clinical care protocols and recovery procedures using variety of delivery methods including didactic learning, simulation based training, workshops, debriefing activities, or role-play to combat WPV in an attempt to provide the most safe and supportive workplace environment, while ensuring delivery of highest quality, safe and compassionate care to their communities (23).

Some institutions even have “zero tolerance” policies against any form of hostility or violence aimed at HCW. The term originated in the 1980s in the context of neighbourhood policing to control crime (24, 25) and is now commonly used in WPV reduction or prevention campaigns and guidelines (5, 19, 25). Overall, zero tolerance policies are used as prevention or deterrence of serious acts of WPV (25) and convey the message that the organization stands firm against WPV, which may contribute to a sense of safety amongst patients and staff (5). However, they also have serious disadvantages in that they tend to address misconduct through strict, automatic sanctions for violations, with little consideration given to individual circumstances (1–3, 26) something that is impractical in most clinical contexts (11). Zero tolerance policies are often viewed skeptically by staff, who challenge the credibility of “zero tolerance” statements when staff continue to see repeated incidents with no clear consequences for perpetrators (5). Zero tolerance policies are also viewed as confusing given their tension with the HCW's duty to care and human rights legislation that protects patient's right to access treatment (5, 25). Despite these efforts to address WPV, the incidence of WPV in healthcare settings continues to escalate leading to a call for action from stakeholders (13, 27, 28).

Our institution, SJHH, is no exception. Box 1 provides some examples of reported WPV incidents. These examples are merely intended to illustrate the extent of the problem faced by increasingly diverse urban core cities like Hamilton, Ontario with increasingly polarized society, increasing social inequities, drug toxicity crisis and increased mental health burden from the impact of COVID-19 pandemic. In alignment with the organization's 2024–25 priorities and with the Joint Board of Governors, Executive Leadership Team along with our affiliated academic institutions McMaster University and Mohawk College are committed to fostering a physically and psychologically safe environment for our healthcare workers, volunteers and learners.

Box 1 **Examples of de-identified reported WPV incidents**

□ Patient requested PRN medication. Writer approached to administer. patient began to say racially inappropriate comments. he received firm limit setting and became sexually inappropriate with writer. Sexually inappropriate behavior continued following interaction with writer.
□ Patient racially inappropriate “shut up, black s*******”
□ Entered client's room to administer medication and client was verbally abusive and told writer that “You belong to be locked away in Africa”
□ Patient at nursing station, asked to go to courtyard. Staff XX declined use of courtyard at this time. Patient verbally escalated. Staff member XX in nursing station. Patient yelling at staff members stating, “Go back home you f****** P***,” “I'll cut your throat you bloody c***!” Patient demanding to call the police. Staff unplugged phone, patient unable to call police. …
□ Changing an aggressive patient. While the patient was trying to hit and kick….

### Objectives

The overall goal of this paper is to describe the methodology for a quality improvement project to advance safety culture at SJHH, an academic Health Sciences Centre, which forms part of the St Joseph's Health System. Safety culture refers to placing a high level of importance on a combination of shared safety beliefs, perceptions, values and attitudes influencing how we do our work particularly by addressing WPV (29). The objectives of the project are: (i) To assess barriers and facilitators of physician reporting of WPV incidents; (ii) to evaluate gaps in existing WPV data and reporting systems (iii) to identify system level opportunities to improve WPV prevention and response; iv) to monitor changes in the WVP related outcomes over time measured by reporting rate, incident outcome and trends and the priorities identified by staff consultations.

### Setting

Affiliated with McMaster University and Mohawk College and located in Southern Ontario, Canada, SJHH is an organisation with about 1,000 beds and bassinets and with an operating budget of around one billion Canadian dollars, close to 5,000 staff, about 1,300 credentialed medical doctors and professional staff and around 7,000 learners, which includes trainees from medical and non-medical graduate and undergraduate programs from our university and college partners. SJHH manages high volumes of admissions, surgeries, ambulatory visits and emergency care. It is among Canada's top research hospitals and is recognized for its renal, urinary programs, robotic surgeries and integrated comprehensive care models.

There are several factors that make SJHH a unique setting for making WPV a priority. Firstly, like most mission based hospitals with a Catholic identity, we are actively designing programs to meet the needs of the most vulnerable patients. Examples of such outreach programs include Youth Wellness Centre in the Downtown Core aimed at providing access to culturally sensitive mental health or addiction care and support to youth aged 17–25 years in a barrierfree, safe space in downtown Hamilton and Seniors’ Mental Health, which provides both in-patient and home care to people living with Alzheimer's and other forms of dementia. This may result in an increased number of our staff visiting patients in their homes, alone, without the security and support of a hospital environment. Second, we support people who are vulnerable, often not feeling respected by society, are fearful or mistrustful of healthcare and live in impoverished circumstances, all factors that may influence responsive behaviours. Third, we serve people with very acute conditions. For example, we have a large kidney program which is important to better serve our community, but creates challenges when nephrology patients are violent (30, 31), but also require about 4 dialysis inductions per week. Managing the burden of dialysis and kidney disease can understandably cause patients to not be at their best. Fourth, as a provider of the second-largest mental health and addiction program in Ontario with about 170,000 mental health visits a year, our HCWs at SJHH face a much higher risk of WPV than a typical healthcare setting. Evidence suggest that HCWs working in psychiatric sectors face one if the highest risks of WPV (32) We offer outpatient and inpatient services to patients with varying acuity of mental health and addictions conditions from mild to very severe. Committed to providing innovative and compassionate care, SJHH is the first organisation in Canada to partner a mental health worker with police when responding to a 911 emergency call. 5) Lasty, our values of dignity, respect, justice and responsibility compel us to treat WPV as a major concern and priority to address for the sake our employees, patients and learners. They deserve to work in an environment that is free from any form of violence.

As an organization, we have been implementing WPV initiatives for a long time e.g., safety incident reports, regular reporting on quality improvement and patient safety indicators, workplace violence prevention committees and requirements from employment legislation. However, evidence from daily reports of WPV incidents suggests that the problem continues and requires specific attention.

## Design/methods

### The Workplace Safety Governance Committee and terms of reference

The President of SJHH set up the Workplace Safety Governance Committee as an advisory body to build on the work of the current operational Prevention of Violence in the Workplace Committee, to meet the goal of SJHH being the most safe and supportive environment for all healthcare workers, learners and volunteers. The Committee includes diverse representation from hospital executive leadership team, Patient Family Advisory Council, Physician Representation, Clinical Directors and Managers, Clinical Ethics, Occupational Health and Safety, Security Services, Communications, Public Affairs & Stakeholder Relations and learners (See Supplementary Appendix A for a template of the terms of reference). The Committee also put together some principles to guide their work (see Supplementary Appendix B). The principles were intended to be a foundation to facilitate respectful, inclusive discussions on a sensitive topic of WPV by a committee comprised of people from diverse cultural and academic backgrounds, experiences and roles in the hospital.

### Methods

**Figure 1:**
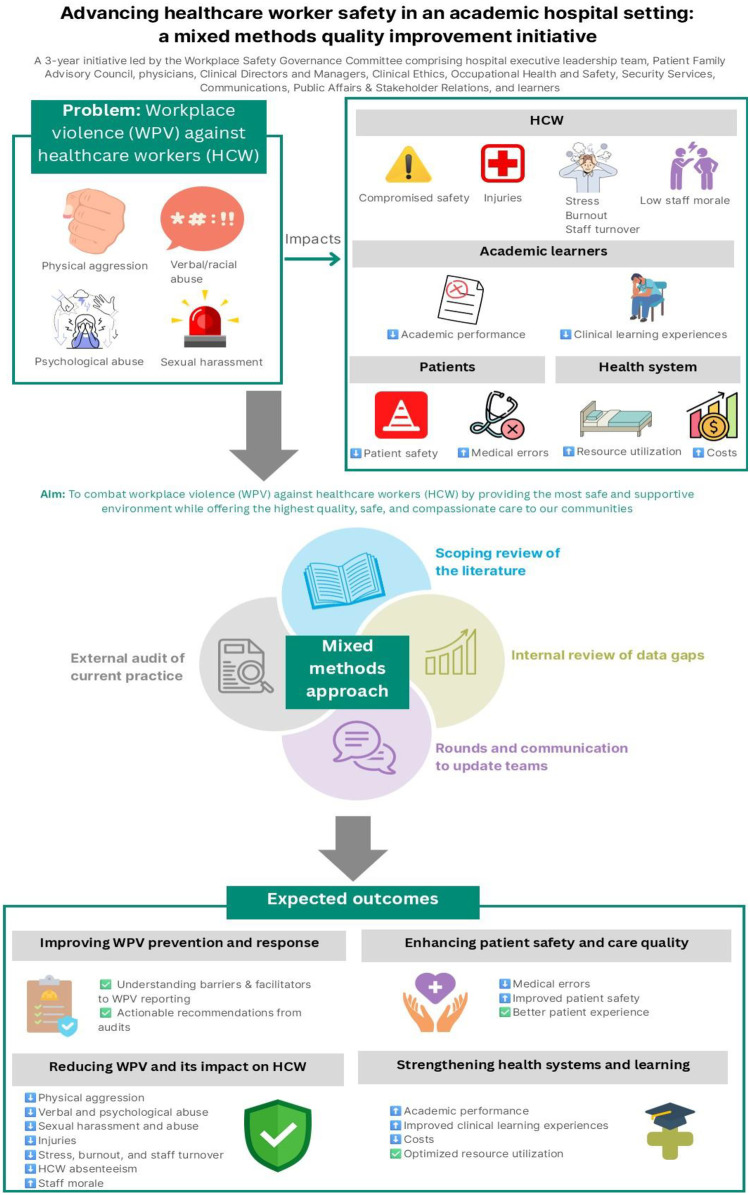
Schematic representation of the mixed methods quality improvement initiative for advancing healthcare worker safety in an academic hospital setting.

The Committee's work is designed as an organization wide quality initiative to be conducted over three years. Rather than a traditional research study or single mixed method design this work will follow a structured quality initiative framework consisting of phases of assessment, gap identification, engagement with stakeholders and making improvements at system level. The methods used will be informed by mixed methods but will be interconnected to support the organizational quality initiative. The following are the integrated components of a quality improvement initiative informed by mixed methods (see Figure 1). These components will provide integrated insights to inform decision making, to support co creating solutions and hence guide organizational change.

(i) *Scoping review*: WPV in healthcare has been extensively documented however the majority of literature on reporting of WPV incidents is focused on nursing staff (15–17) and there is limited literature on reporting behaviours by physicians or doctors. Our own internal data reports also show that a high proportion of WPV incidents are reported by nurses and other frontline staff, while only a small proportion reported by physicians. For the calendar year January to December 2024, out of a total of 1,066 workplace violence incidents reported at SJHH, approximately 66.1% were reported by nursing staff, while only 0.8% were reported by physicians and residents. As noted earlier, our success in finding solutions to address WPV depends on full reporting of WPV incidents by all those affected by the violence including physicians. Under reporting by physicians may result in incomplete data hence fragmented understanding of workplace safety, limiting the effectiveness of institutional responses to WPV. To address this gap, we will conduct a scoping review in accordance with the Preferred Reporting Items for Systematic Review and Meta Analysis Extension for Scoping Review (PRISMA-ScR) guidelines. A scoping review methodology is identified for this study as this is an emerging topic and we need to map the existing evidence to clarify the concepts and identify gaps. The review will focus on studies related to physicians and medical learners in healthcare settings and examine both formal and informal reporting practises (33). The aim is to determine the barriers and enablers of WPV incident reporting by physicians in order to inform policy development and institutional practises to enhance reporting and hence workplace safety. Please refer to Table 1 for details of the scoping methodology.
(ii) *External Audit and Feedback*: Audit and feedback is commonly strategy used in healthcare process improvement either on its own or as a component of multifaceted quality improvement strategies (34). We will engage the Institute for Healthcare Improvement (IHI) as an external partner with expertise in WPV and patient safety to perform an audit of our current practices and structures and to identify areas for improvement. IHI will conduct an independent audit of SJHH's current practises, governance structure and foundation using established, evidence-based frameworks and assessment methods (Refer to Table 2 for details of the IHI audit framework). The work will be done in three phases, first will be a pre diagnostic visit to perform an initial scan of safety and workforce well being to help define the scope of full diagnostics. Phase 2 will be the full diagnostic visit, data collection, review of information and on-site comprehensive assessment. Finally, all the findings will be presented as recommendations with an initial coaching for implementation of the improvement strategies. Founded in 1991, IHI is a nonprofit global and leading organization committed to improving health and healthcare worldwide (35). Throughout the initiative findings from IHI audit will be used as data for organizational learning, supporting psychological safety and open engagement. The expected outcome of the audit is to work on an improvement roadmap to guide priorities and decisions impacting patient and workforce safety. The audit findings will serve as a baseline external assessment identify the strengths, gap and hence priorities. The recommendations from the audit will be mapped along side internal data and qualitative findings from the leadership rounds to support the evidence. These findings will help prioritize and refine improvement strategies and will be used to assess progress over time within the quality improvement initiative.
(iii) *Review of data gaps*: As an academic health sciences centre, using data to guide decisions is an integral part of our system. To strengthen our capacity to respond effectively to workplace violence incidents, we will be reviewing the data variables currently collected within our system. This review will identify any data gaps and determine what additional information could support more informed, evidence based decisions and corrective strategies. There is concrete evidence that identifying and bridging data gaps along with benchmarking data collection metrics gives us the ability to determine additional information required to help with continuous improvements within the organization. We will engage the Workplace Safety and Insurance Board to perform an internal review of SJHH data on mental stress injuries related to WPV reports with similar organizations to identify any gaps on data. WSIB is one of the largest insurance organizations, covering over five million people in more than 300,000 workplaces across the province of Ontario, Canada, WSIB provides no fault collective liability insurance and access to industry-specific health and safety information (36).
(iv) *Qualitative discussions and rounds for regular updates and communication*: We will use qualitative descriptive approach consistent with established qualitative methodology and quality improvement reporting guidance, to explore staff perceptions on WPV across the hospital setting, using ‘structured rounds' method. The units will be selected based on the review of preceding 2 year WPV incident data to identify the priority areas across acute care, mental health and off site services. All staff on the units will be invited to ensure diverse participant group including support and leadership role. Participation will be voluntary. The number and frequency of rounds will be flexible with multiple sessions across units over course of the three years, this will be informed by emerging findings and organizational priorities. The rounds will be conducted as an exploratory process to elicit concerns and advance open communication about WPV and to generate ideas on how to collaboratively tackle the problem (37). These will be facilitated by a quality consultant in collaboration with workplace safety governance committee, EDI (Equity, Diversity and Inclusion) and hospital leadership team to ensure consistency and equitable participation across different roles and units. The data will be collected as detailed field notes and structured group discussion summaries. The data will be de identified at all individual levels and organized by units and date for comparison across different settings. Analysis will follow a thematic approach, to identify recurring patterns, key concerns, and potential solutions. Findings will be compared across different clinical contexts (e.g., acute care, mental health, and off-site services) to identify both shared and context specific issues. We will share the findings internally using platforms that include newsletters, management forums, corporate committees, townhall meetings and ‘Open Mike’ where the hospital president shares regular updates and discussion on topical issues. We will also use policy briefs to share updates with the hospital board and other regional partners. The findings will be disseminated externally through quality initiative publications. Please refer to Table 3 for details.

**Table 1:** Summary of scoping review methods details^1.^

Review section	PRISMA-ScR requirement	Description
Protocol and registration	Statement on existence of a protocol; access details; registration name and number	This scoping review will be conducted in accordance with the Preferred Reporting Items for Systematic Reviews and Meta-Analyses Extension for Scoping Reviews (PRISMA-ScR). A study protocol will be developed and registered with the Open Science Framework.
Eligibility criteria	Explicit inclusion/exclusion criteria (population, concept, context, years, language, publication status) with rationale	This scoping review will include peer-reviewed, English-language empirical studies published from January 1, 2015 to the date of the search. Eligible studies must involve human participants aged 19 years or older and be conducted in healthcare settings, including but not limited to hospitals, emergency departments, clinics, primary care practices, or other medical or clinical workplaces. The population of interest comprises physicians and medical learners, including staff physicians, specialists, surgeons, general practitioners, fellows, residents, medical students, and other medical trainees. Studies involving broader healthcare worker samples will also be eligible provided that physicians and/or medical learners are explicitly included in the study population. The phenomenon of interest is workplace violence directed toward healthcare personnel, encompassing verbal abuse, threats, harassment, bullying, aggression, and physical or sexual assault occurring in the work environment. Included studies must address the reporting of workplace violence and/or factors related to reporting, such as barriers and facilitators, attitudes, perceptions, practices, under-reporting, incident reporting behaviors, whistleblowing, or reporting intentions. Quantitative, qualitative, mixed-methods studies, as well as systematic or scoping reviews that report primary or synthesized data relevant to this phenomenon, will be considered for inclusion.
Information sources	Full list of databases searched, date ranges, grey literature sources, hand-searching, expert contact, and date of last search	The literature search for this scoping review will be conducted in the electronic databases MEDLINE (via PubMed) and Embase (via Elsevier). Both databases will be searched from January 1, 2015 to the date of the final search to capture contemporary evidence reflecting current workplace policies and reporting systems. In addition to database searching, relevant grey literature will be identified through targeted searches of organizational and governmental websites, professional medical associations, and patient safety or occupational health agencies, as well as through Google Scholar using predefined search terms. To ensure comprehensive coverage, the reference lists of all included studies and relevant reviews will be hand-searched for additional eligible publications. Where appropriate, experts in the field may be contacted to identify unpublished or ongoing studies relevant to the review topic. The final search date will be documented and reported in the completed review.
Search strategy	Reproducible full electronic search strategy for ≥1 database, including limits	Search: ((((((((((risk*) OR (predictor)) OR (determinant)) OR (habit)) OR (incident reporting)) OR (barrier*)) OR (facilitator*)) OR (associate*)) AND ((((“work-related violence”) OR (“physician workplace violence”)) OR (“violence against physician*”)) OR (“work related violence”))) AND (((((((((violence) OR (aggression)) OR (harass*)) OR (abuse*)) OR (assault*)) OR (“anger-related”)) OR (“anger related”)) OR (hostil*)) OR (“occupational violence”))) AND (((((((((Physician*) OR (doctor*)) OR (specialist*)) OR (surgeon*)) OR (“medical practitioner”)) OR (“medical student”)) OR (fellow*)) OR (“medical learner”)) OR (trainee*)) Filters: in the last 10 years
Selection of sources of evidence	Description of screening process (title/abstract screening, full-text review, number of reviewers, conflict resolution	All records identified through the database and grey literature searches will be imported into COVIDENCE, and duplicates will be removed prior to screening. Titles and abstracts will be independently screened by two reviewers against the predefined eligibility criteria. Studies deemed potentially relevant by either reviewer will be retrieved for full-text review, which will also be conducted independently by the same two reviewers. Any discrepancies or disagreements at either the title/abstract or full-text screening stages will be resolved through discussion and consensus. If consensus cannot be reached, a third reviewer will be consulted to adjudicate and make the final decision regarding study inclusion. The study selection process will be documented and reported using a PRISMA-ScR flow diagram, detailing the number of records identified, screened, assessed for eligibility, and included in the review.
Data charting process	How data were extracted (charting form, pilot testing, duplicate extraction, reviewer independence)	Data charting will be conducted using a structured form to systematically extract relevant information from included studies. The form will capture study characteristics, healthcare settings, physician participant details, definitions and types of workplace violence, reporting mechanisms, rates of reporting (if available), and identified barriers to reporting, as well as key recommendations. The charting form will be pilot tested by two independent reviewers and refined iteratively. Data extraction will be performed independently by two reviewers, with discrepancies resolved through consensus or consultation with a third reviewer.
Data items	Defined list of variables extracted (e.g., study design, setting, barriers, enablers) and assumptions	Study characteristics (e.g., study design, year of publication, country) Healthcare setting (e.g., hospital, emergency department, outpatient or community settings) Type and definition of WPV (e.g., physical violence, verbal abuse, threats, harassment) Reporting mechanisms (e.g., formal incident reporting systems, informal reporting) Barriers to reporting (e.g., time constraints, normalization of violence, fear of repercussions, lack of feedback) Outcomes related to reporting (e.g., reporting rates, perceived usefulness, organizational response)
Critical appraisal of sources	Rationale for appraisal (if done), methods, and use in synthesis	Not applicable
Synthesis of results (methods)	Explanation of how data will be summarized (narrative synthesis, thematic analysis, mapping)	The results will be synthesized using descriptive numerical analysis, narrative synthesis, and thematic analysis. Study characteristics (e.g., design, setting, physician population, and type of WPV) will be summarized to describe the scope and distribution of the evidence. Key findings will be integrated through narrative synthesis, and barriers and enablers to WPV incident reporting by physicians will be identified and grouped into overarching themes across individual, professional, organizational, and system levels. Findings will be mapped to highlight evidence gaps and inform future research and practice.

^1^ Adapted from PRISMA-ScR checklist: academic medicine.

**Table 2:** Summary of Institute of Healthcare Improvement audit methods details.

Framework/Tool	Purpose	How IHI Will Use It in the Diagnostic Gap Analysis
Leading a Culture of Safety: A Blueprint for Success	Strengthens leadership behaviors, governance structures, and cultural foundations that support safety.	The Institute for Healthcare Improvement will use this framework to assess how consistently leaders model safety expectations, how Just Culture principles are applied in practice, and how leadership alignment influences the overall safety climate at SJHH.
Safer Together: A National Action Plan to Advance Patient Safety	Defines four foundational domains for patient and workforce safety: Culture, Leadership and Governance; Patient and Family Engagement; Workforce Safety and Well Being; and Learning Systems.	IHI will evaluate SJHH's maturity across the four domains to identify systemic gaps that may be limiting progress. Results from the Safer Together Online Team Self-Assessment Tool will be analyzed to understand how staff across roles perceive safety culture, readiness, and organizational priorities.
Framework for Standardized Data Collection of Workplace Violence Incidents in Health Care	Provides a structured method for classifying, documenting, and analyzing workplace violence (WPV) incidents.	IHI will compare SJHH's current WPV reporting practices against this framework to identify gaps, such as incomplete capture of psychological harm, other incident types, and follow-up or learning processes.
IHI Framework for Safe, Reliable, and Effective Care	The framework is built on two foundational domains—culture and a learning system—that work together to support safe, reliable, and effective care. It defines nine interrelated components with practical strategies, centers patients and families as the driving force, and includes a diagnostic tool to help organizations assess and strengthen their implementation.	IHI will use this framework to conduct a diagnostic gap analysis for workforce safety by evaluating how effectively organizational culture and the learning system support staff physical and psychological safety across the nine components. This will identify gaps between current and desired practices, guiding prioritized actions that will strengthen workforce safety, engagement, and overall system reliability.
Qualitative and Operational Assessment Methods (interviews, focus groups, safety rounding, direct observation)	Captures the lived experience of staff and uncovers cultural or operational dynamics not visible in formal data.	IHI conduct interviews and hold meetings with focus groups including Joint Health and Safety Committees, union partners, and the PVW Committee to validate findings from formal frameworks, deepen understanding of caregiver experiences, and identify cultural or workflow issues that may not be captured in existing policies or performance metrics.

**Table 3:** Summary of qualitative methods details^2.^

Item	Description	How we will address each methods item
Qualitative approach & paradigm	Specify the qualitative approach (e.g., qualitative descriptive, participatory action research) and guiding paradigm (e.g., constructivist, critical, equity-oriented).	Qualitative descriptive study design approach will be applied to explore staff perception of WPV. Discussion rounds will be conducted across hospital units as an exploratory and iterative process to elicit concerns, support open communication, and co-generate strategies to address WPV. This approach was selected so we can capture participants insights to generate actionable insights to inform organizational decision-making and policy development.
Researcher characteristics & reflexivity	Identify who conducts the rounds, their roles, training, and positionality; describe reflexive steps to address power and racial dynamics.	Discussion rounds will be conducted in collaboration with Workplace Safety Governance Committee, EDI (Equity, Diversity, and Inclusion) team, and hospital leadership team, facilitated by quality consultant for consistency
Context	Add country, health system type, hospital characteristics, and organizational context related to WPV and EDI.	St. Joseph's Health Care Hamilton is a large academic health sciences organization in Hamilton, Ontario, Canada. The organization provides specialized mental health, rehabilitation, complex care, and community-based services across multiple sites. Like many health care institutions, it faces ongoing challenges related to workplace violence, particularly in mental health and high-acuity settings, and has an established organizational commitment to EDI that informs its approach to WPV prevention and response.
Sampling strategy	Explain how units are selected, who is invited, inclusion/exclusion criteria, voluntary participation, and how diversity is ensured.	WPV incident data from the previous two years will be reviewed to identify the five priority areas within different service areas. This will then be used inform the selection of units for conducting the discussion rounds. The sample will include units across three main service areas: acute care, mental health, and off-site. All employees working in the samples units will be invited to the discussion rounds. Over time, the number of sessions will be flexible guided by the themes that emerge during the rounds. Rounds will be facilitated and structured to promote equitable participation across roles ensuring equal participation from managerial, non-managerial and support services staff.
Ethical issues	State REB approval/exemption, consent process, confidentiality protections, and psychological safety considerations.	This is not applicable because this process is considered part of a quality control process by the hospital
Data collection methods	Details of data collection procedures analysis iterative process	The data that are generated during the discussion rounds will include detailed notes, summaries of group discussions to document key themes, factors contributing and proposed interventions. The data will be analysed iteratively after every discussion round to allow the team to adjust the prompts for discussions for subsequent meetings and also explore more emerging themes in alignment with institutional priorities.
Data collection instruments & technologies	Specify discussion guides, prompts, facilitation tools, and whether audio recording or field notes are used	For consistent data collection, we will create an open-ended questions tool to guide conversations in order to provide frontline healthcare workers opportunity to raise the issues. Detailed field notes will be collected during the sessions that will be summarize under the emerging themes. We will also document any relevant studies or protocols that are brought forward during the discussions.
Units of study	Provide numbers of rounds, participants, roles, and relevant demographic characteristics (as appropriate).	Units will be selected to reflect various clinical functions, patient population, staff roles, and exposure to WPV, including both acute care, mental health and off-site groups. Discussion rounds will typically take place during routine unit activities and will include available staff.
Data processing	Explain transcription, note synthesis, anonymization, data storage, and preparation for analysis.	Information collected during the rounds will be organized and securely stored with no personal information of any individuals; the information will be stored for the unit only (for example Clinical teaching unit – notes/ summary/ themes). Data will be organized by unit type and date to support review and comparison. A quality consultant will support data processing by helping to organize, review, and summarize the information collected, ensuring consistency and completeness across units.
Data analysis	Describe analytic method (e.g., thematic analysis), coding process, analyst roles, software (if any), and theme development.	Data will be analyzed using a qualitative thematic approach to identify recurring patterns, key concerns, and proposed solutions related to workplace violence. Analysis will occur as data are being collected to inform subsequent rounds. Findings will be compared across acute care, mental health, and off-site settings to identify shared and context-specific issues.
Trustworthiness techniques	Add rigor strategies: triangulation, member checking, peer debriefing, audit trail, or reflexive journaling.	Data will be collected across multiple units and settings to capture diverse perspectives and support credibility through variation. An iterative process of data collection and analysis will allow emerging findings to be explored and refined. Analytic discussions within the study team, supported by a quality consultant, will promote consistency and reflexivity. Use of standardized discussion guides, field notes, and documentation will support transparency of findings.
Dissemination (not Methods)	Move this content to a separate “Knowledge Translation” or “Dissemination” section; do not include in Methods per SRQR	We will share the results of the findings internally and publicly using quality improvement publications in QI scientific journals

^2^ Adapted from PRISMA-ScR checklist: academic medicine.

Our approach is consistent with other Canadian quality improvement frameworks that use structured, system level quality initiatives approaches to address safety concerns across health systems (38).

### Outcomes

We will report the findings on the barriers and facilitators of physician reporting of WPV incidents. We will also report on WPV data gaps from our internal review. We will collect institutional data on the following outcomes that include the number of reported WPV incidents at SJHH of (i) *physical aggression* (e.g., pushing, punching, scratching, spitting, hitting, kicking, using a weapon, throwing objects, or other forms of physical harm); (ii) *verbal abuse* (e.g., threats, shouting, or derogatory language); (iii) racial abuse (i.e., Racial insults), (iv) *psychological abuse* (e.g., intimidation or coercive behavior); (v) *sexual harassment or assault* (e.g., unwanted advances or inappropriate behavior) and vi) *Code White*. The reporting will be stratified by physician, nonphysician, learner and clinical unit under each category, with no individual details included. We will also report the number of *lost time days* because of WPV incidents. Where possible, we will also describe any lessons learned and suggestions for solutions from qualitative discussions with frontline workers and including findings and recommendations from the IHI audit.

### Timeframe

We anticipate that this project will take three years to complete, with the first year dedicated to learning more about what WPV looks like in our institution and how we can best address it.

### Analysis

The analysis will be primarily descriptive and aligned with the quality improvement approach. We will be monitoring changes over time to baseline data. Baseline data will be obtained from prior two years as reference. The quantitative data will be summarized using counts and percentages for continuous variables and means (standard deviation) or median (first quartile, third quartile) for categorical variables as appropriate. Primary outcomes will include WPV incident reporting rates (with particular attention to physician reporting), overall incident trends, and lost time days associated with WPV. Secondary outcomes will include types of WPV incidents, incident trends based on the units/departments, identification of data gaps in reporting system, barriers and enablers of reporting, HCW perception of workplace safety and learning from the rounds.

Process charts will be used to display trends over time and to assess variation and signals of change in key indicators. Interpretation will focus on identifying meaningful changes in patterns and progress over time, consistent with quality improvement methodology, rather than formal hypothesis testing. Qualitative data will be synthesized using a thematic approach to identify recurring patterns, lessons learned, and actionable insights related to barriers, facilitators, and staff experiences. Findings from quantitative and qualitative analyses will be integrated to support interpretation and inform ongoing, evolving refinement of the initiative.

### Ethics

This is a quality improvement project which is exempt from research ethics approval under the Tri-council Policy Statement (39) which provides ethical standards for conducting research with humans in Canada.

### Dissemination

This will be a 3-year quality improvement initiative project. The results and findings will be shared internally and externally to allow reflections on lessons learned and to continuously improve safety processes and procedures within the hospital. We have several stakeholders as audiences for the findings of this initiative. They include hospital employees or HCW, hospital board members, the executive leadership team, local university partners, researchers, policymakers, local and regional healthcare partners, the media/press, the general public and other healthcare professionals. We will use different platforms for disseminating the results, targeted appropriately for each stakeholder**.**

#### Internal communication platforms

As part of Method iv, we will share the findings internally using platforms that include newsletters, management forums, corporate committees, town-hall meetings and ‘Open Mike’ where the hospital president shares regular updates and discussion on topical issues.

#### Peer-reviewed publications

The study protocol and results will be published in open access peer reviewed scientific journals. We will follow appropriate recommended reporting guidelines for quality improvement initiatives (34).

#### Presentations

We plan to present the study findings at appropriate national, regional and international meetings. These are usually attended by other epidemiologists, researchers, policymakers and health care professionals.

#### Engagement and policy/issue briefs

All findings will be shared with the hospital board and other regional partners.

#### Social media platforms

Social media have a far reach and therefore we will use platforms such as X (formerly Twitter), LinkedIn, Instagram and Facebook to share key messages from our findings.

#### Press release

We will also explore ways to share the results with a wide community via press releases.

## Discussion

WPV against HCW is a complex and persistent challenge in health care, it presents a major barrier to achieving our collective goal of good health outcomes for both patients and HCW. This is our first attempt to put together additional methods to better understand the extent and nature of the problem in our setting, to develop evidence-based strategies to mitigate or manage it. This work provides a model of system level quality improvement initiative where multiple methods will be systematically used over a three year period within an evolving framework to inform organizational change, rather than representing a single unified research study design. We anticipate that this 3year work will include many (un)foreseen challenges, but it will also provide useful information to enrich our learning and inform our next efforts to address WPV. The scoping review will address the gap by examining the reporting enablers and barriers among physicians as there is evidence from recent studies (40) that physicians, residents and medical students often under report WPV incidents, this masks the extent of the issue and limits comprehensive care team based response.

Further engaging an external review/audit will provide an objective and comprehensive overview of SJHH current practices that are relevant to workforce safety and psychological wellbeing. We are hoping this will help identify systemic causes contributing to WPV and hence opportunities of improvement. This will also help us pinpoint tools, processes and develop frameworks to manage and address disruptive behaviours. The unbiased approach further enhances the credibility of finding and help engage external and internal stakeholders with confidence.

In depth review of both qualitative and quantitative data for SJHH will provide us with a deeper understanding of current state, give us an opportunity to identify key performing leading and lagging indicators as per the industry benchmarking standards. We also aim to identify key high risk programs/units and apply indicators to assess the effectiveness of current strategies and resource allocation (41).

Our methodological approach is robust, using mixed evidence-based methods for data collection from different sources that include scoping review of the literature, engagement of internal and external stakeholders and audit of our current practices and standards. These outcomes will inform improvements of WPV related policies and procedures, streamlined processes for incident reporting, improved risk identification, strengthened post incident response and support mechanisms.

## Group member of The Workplace Safety Governance Committee

President (Co-Chair) – MH; VP Research (Co-Chair) – LT; EVP Clinical Operations and Chief Nursing Executive – BC; EVP People, Culture & Strategy – CF; Patient Family Advisory Council member – SM-B; Physician Representation – CR; Clinical Director – KA-B; Clinical Manager – CB; Clinical Ethicist – LB; Director Occupational Health and Safety – SS; VP Communications, Public; Affairs & Stakeholder Relations – CW; Manager, Education - AP; Manager, Security Services – CP.

## Supplementary material

Supplementary Appendices A & B Workplace Safety Governance Steering Committee (A) Template for Terms of reference and (B) Guiding principles for the committee work.
