# Supplementary material for: Advancing healthcare worker safety in an academic hospital setting: a mixed methods quality improvement initiative protocol
Source: Front Health Serv. 2026 May 1;6:1676416. doi: 10.3389/frhs.2026.1676416 (PMC13176298; doi:10.3389/frhs.2026.1676416)
Supplement: Supplementary Appendices A & B — Workplace Safety Governance Steering Committee (A) Template for Terms of reference and (B) Guiding principles for the committee work. [file Supplementaryfile1.docx]

**Appendix A: TEMPLATE FOR WORKPLACE SAFETY GOVERNANCE STEERING COMMITTEE AT AN ACADEMIC HEALTH SCIENCE CENTRE**

**TERMS of REFERENCE**

Date: mm/dd/yyyy

**GOAL:** For Hospital X to be a safe and supportive environment while providing the highest quality, safe and compassionate care to the communities it serves.

**PURPOSE:** The Workplace Safety Governance Committee will serve as a governance and advisory body to the Hospital President, Board of Governors and Executive Leadership Team to meet the goal of Hospital X being the most safe and supportive environment for all healthcare workers, learners and volunteers.

**OBJECTIVES**

- To govern the organization’s goal of improved healthcare worker, learner and volunteer safety by mitigating risks of workplace violence – including physical, verbal, racial, psychological and sexual violence.
- To advocate for a culture of safety – both physical and psychological.
- To raise awareness of workplace safety and the impacts of violence in the workplace, both internally and externally.
- To seek ways to ensure actions to address workplace safety are reflective of the needs of patients and families and lives the mission and values of Hospital X.
- To utilize the principles of co-design with different hospital groups and ensure operational excellence in the provision of strategies and initiatives addressing workplace safety.
- To advocate to government for further protection of all healthcare workers, learners and volunteers.

**RESPONSIBILITIES**

- Review the literature of health care workplace violence, understanding best practices nationally and internationally.
- Support external assessment of current practices in relation to workplace violence prevention and the intersection with patient safety.
- Explore opportunities with regional bodies to achieve Workplace Safety Excellence designations.
- Support the development and implementation a 3-year action plan to help prevent physical, verbal, psychological, sexist and racist violence towards our healthcare workers, learners and volunteers.
- Support the development of a performance measurement plan aligning with the action plan.
- Support the development of communication and stakeholder relation plans aligning with the action plan.

**MEMBERSHIP: Diverse** representation from hospital executive leadership team, Patient Family Advisory Council, Physician Representation, Clinical Directors and Managers, Clinical Ethics, Occupational Health and Safety, Communications, Security Services, Public Affairs & Stakeholder Relations, learners

**RESOURCE:** (Under)graduate student

**ACCOUNTABILITY and REPORTING:** The Workplace Safety Governance Steering Committee is accountable to the Hospital Board through the Co-Chairs. The committee shall report to the Board HR Committee and Quality Committee on a quarterly basis.

**MEETING FREQUENCY:** Meetings will be held 5 times per year. It is the expectation that all committee members will attend meetings as requested. In the event that members are unable to attend they will notify the Co-Chairs of their absence and advise who will be attending in their absence. Alternates or delegates must be approved by one of the Co-Chairs. Ad-hoc attendees will be invited as needed.

**COMMITTEE DECISION PROCESS**

- Information: the committee will ensure clear, consistent and accurate information is used to support decisions.
- The committee shall strive for consensus leading to unanimity; in the event that consensus cannot be achieved the Co-Chairs shall make the final decision. If the Co-Chairs are not in agreement with the consensus opinion of the committee, they can supplant a committee decision with their own decision, so long as they clearly communicate the rationale.
- Quorum for meetings shall be 50% of the membership plus one.

**COMMUNICATION:** Agendas to be provided to committee four days prior to meeting date. Minutes inclusive of action items and decisions will be provided to all Committee members within 2 weeks of the meeting date and recirculated with the next meeting agenda.

**TERMS OF REFERENCE REVIEW:** The terms of reference (TOR) shall be reviewed annually to ensure they up to date and relevant

**Appendix B: GUIDING PRINCIPLES FOR WORKPLACE SAFETY GOVERNANCE COMMITTEE WORK**

| **Principle** | **Explanation** |
| --- | --- |
| **Commitment to a Safe Culture** | We are dedicated to fostering a culture of safety, transparency and mutual respect. |
| **Commitment to anti-discrimination / anti-oppression /anti-racism, anti-stigma** | We are committed to fostering anti-stigma, anti-discrimination, anti-oppression and anti-stereotypes to challenge ideas that certain populations are inherently more violent than others. |
| **Understanding Medical Conditions** | While medical conditions may provide explanations for some incidents, they are not an excuse for violent behavior. Cognitive impairments or language barriers, for example, can help explain challenging behaviors. |
| **Patients can teach us to be better** | There are reported incidents that show violence is not the patients’ fault and we can put systems in place to help both us and patients improve. |
| **Evidence-Based Approach**: | Our approach will be guided by research, lived experiences, regulatory guidance, industry standards and best practices. |
| **Commitment to Collaboration** | We are dedicated to working with all staff, particularly frontline workers, to regularly assess risks, hazards and safety policies to inform and develop mitigation strategies. |
| **Encouraging Open Communication** | Open communication and reporting of safety incidents are strongly encouraged, with a commitment to learn and address any barriers to reporting at all levels. |
| **Data-Driven Improvement** | We commit to analyze data and trends to continuously improve safety practices. |
| **Sharing Knowledge** | Experiences and lessons learned will be shared with broader hospital communities through academic publications, supporting the hospital’s academic mission |
| **Year of Discovery** | We are committed to discovery on our current practices, different perspectives on strategies, gaps in knowledge including ways to address the gaps. It will take about 3 years to complete our work. |
| **Building trust** | We will work to earn the trust of staff, patients and learners through transparency, compassion and accountability |
